# Prospective Evaluation of Clinical and Laboratory Profiles of Febrile and Afebrile Immunosuppressed Patients Presenting to the Emergency Department

**DOI:** 10.3390/medicina61050889

**Published:** 2025-05-14

**Authors:** Tuğrul Topal, Esra Pamukçu, Muhammet Gökhan Turtay, Gülşen Yalçın, Harun Kürşat Şahingil, Mehmet Sezer

**Affiliations:** 1 Emergency Medicine Clinic of Necip Fazıl City Hospital, 46050 Kahramanmaraş, Turkey; 2 Department of Statistics, Faculty of Science, Firat University, 23119 Elazığ, Turkey; 3 Emergency Medicine Clinic of Konya City Hospital, 42020 Konya, Turkey; 4 Department of Medical Biochemistry, Turgut Ozal Medical Center, Inonu University, 44210 Malatya, Turkey; 5 Emergency Medicine Clinic of Yeşilyurt Hasan Çalık State Hospital, 44920 Malatya, Turkey; 6 Emergency Medicine Clinic of Besni State Hospital, 02300 Adıyaman, Turkey

**Keywords:** immunosuppressed patients, fever, emergency department, early diagnosis

## Abstract

*Background and Objectives*: Immunosuppressed patients are at higher risk of delayed diagnosis and atypical presentations in the emergency department (ED), requiring prompt management. This study compares febrile (≥37.5 °C) and afebrile (<37.5 °C) immunosuppressed patients admitted to the ED regarding clinical and laboratory parameters, including blood and urine tests, vital signs, final diagnoses, outcomes, and mortality. *Materials and Methods*: Eighty immunosuppressed patients aged 18–82 were prospectively evaluated from May 2019 to May 2020. Data on blood and urine tests, final diagnoses, outcomes, and mortality were recorded using a standardized form. *Results*: Among the 80 patients, 44 (55%) were female and 36 (45%) were male, with a mean age of 58.5 ± 14.72 years. The febrile patients showed higher admission levels of lactate dehydrogenase (LDH), interleukin-6 (IL-6), procalcitonin (PCT), and longer hospital stays than the afebrile patients. Mortality correlated with low albumin, oxygen saturation, platelet count, and total protein levels and elevated PCT and lipase levels. ICU admissions were linked to low albumin, total protein, and systolic blood pressure levels and elevated LDH, blood urea nitrogen, neutrophil count, and PCT levels. The fever status (febrile versus afebrile) had no significant relationship with the immunosuppression type, complaints, diagnoses, outcomes, or mortality. Final diagnoses varied by immunosuppression type: cholangitis in liver transplant recipients, respiratory infections in cancer patients, and urinary tract infections in kidney transplant recipients. *Conclusions*: Immunosuppressed patients can present with severe conditions, even without fever. Based on our findings, our study emphasizes that measuring PCT in immunosuppressed patients presenting to the emergency department with various complaints but without fever may help reduce the risk of delayed diagnosis.

## 1. Introduction

Immunosuppressed patients represent a growing population in modern medicine, primarily due to advancements in immunomodulatory therapies for organ transplantation, cancer treatments, and autoimmune diseases. While these therapies have significantly improved survival rates and quality of life, they leave patients vulnerable to infections and atypical presentations of diseases [1]. One of the most critical symptoms in this population is fever, often indicating an underlying infection or other serious pathology. However, the immunosuppressive condition can obscure classic infection symptoms, leading to delays in diagnosis and treatment [2]. Unlike individuals with intact immune systems, immunosuppressed patients may exhibit nonspecific symptoms or even afebrile sepsis without the typical inflammatory response [3]. Studies have highlighted the importance of identifying reliable clinical and laboratory markers to aid early diagnosis and prognosis in this vulnerable group [4,5].

Emergency departments (EDs) frequently serve as the first point of contact for these patients, where timely identification of the underlying cause is critical. Delayed recognition of infections or sepsis can lead to rapid deterioration and increased mortality rates. Early recognition of sepsis, prompt initiation of antibiotics, and effective triage are essential to improve outcomes for these high-risk patients [5,6,7]. Although biomarkers such as procalcitonin (PCT), interleukin-6 (IL-6), and C-reactive protein (CRP) have been extensively studied for the diagnosis of infection and sepsis [8,9,10,11,12,13], their clinical performance in immunosuppressed patients remains less clearly defined [14]. In particular, the diagnostic challenge increases in afebrile presentations, which are not uncommon in this population and may lead to delayed antibiotic therapy and worse outcomes [15]. Furthermore, only a limited number of studies have investigated biomarker-guided risk stratification during emergency department (ED) presentation [9,16]. Most of the available literature focuses either on critically ill patients in intensive care settings [10,15] or long-term prognostic outcomes in cancer or transplant patients [11,17]. Additionally, recognizing unique patterns of infection and disease progression specific to immunosuppressed individuals can help guide the development of personalized treatment strategies, potentially reducing morbidity and mortality in this vulnerable group.

This study compares febrile (≥37.5 °C) and afebrile (<37.5 °C) immunosuppressed adult patients—including organ transplant recipients, oncology patients under treatment, and individuals receiving chronic immunosuppressive therapy—presenting to the emergency department in terms of their clinical profiles, laboratory parameters, final diagnoses, patient outcomes, and mortality. The primary hypothesis focuses on identifying differences between these two groups, while secondary hypotheses explore the relationships between clinical and laboratory findings across subgroups defined by immunosuppressive conditions, infection patterns, and outcomes such as ICU admission and mortality. As a result, an enhanced understanding of infection patterns and disease progression in immunosuppressed patients may lead to more personalized treatment strategies, ultimately reducing morbidity and mortality in this high-risk population.

This study aims to contribute to this limited body of research by comparing febrile and afebrile presentations in immunosuppressed patients presenting to the ED and evaluating the utility of various biomarkers in risk stratification.

## 2. Materials and Methods

### 2.1. Study Design and Data Set

In this study, immunosuppressed patients aged 18–82 who were admitted to the Inonu University Turgut Ozal Medical Center Adult Emergency Department between August 2019 and August 2020 in Turkey were prospectively analyzed.

The inclusion criteria included adult patients (aged 18 years and older) presenting to the emergency department who had documented immunosuppression, defined by one or more of the following:

– A history of solid organ transplantation (e.g., liver or kidney transplantation) and current immunosuppressive therapy;
– Diagnosis of malignancy (solid tumors or hematologic cancers) and receiving chemotherapy, radiotherapy, or immunosuppressive therapy within the past 6 months;
– Chronic immunosuppressive medication use for autoimmune or inflammatory diseases (e.g., corticosteroids, biologic agents);
– Primary immunodeficiency disorders (e.g., congenital immune deficiency).

Immunosuppression status was confirmed based on medical records, patient history, and ongoing treatment regimens.

The exclusion criteria disqualified individuals if they met any of the following conditions: being a healthy individual, lacking an immunosuppressive condition, or being under the age of 18.

A patient follow-up form was created for the patients included in the study. The form included the patient’s name, surname, file number, complaints, history, vital signs, systemic physical examination findings, blood and urine tests, radiological findings, outcome status, length of hospital stay, and final diagnosis information. Blood and urine samples were collected at the time of emergency department admission, immediately after triage and initial vital sign assessment, without requiring prior fasting conditions. The outcome was recorded as either admission to the ward or intensive care unit, and the death status was indicated as “present” or “absent”. The data were anonymized, organized, and then subjected to statistical analysis.

Ethical approval for the study was obtained from the Inonu University Scientific Research and Publication Ethics Committee (Decision Date: 4 December 2018; Decision No.: 2018/22-28). The study adhered to the principles outlined in the Declaration of Helsinki.

### 2.2. Definition of Immunosuppression and Subcategorization

Patients presenting to the emergency department were identified as immunosuppressed based on their medical history and anamnesis forms. The patients were categorized according to immunosuppression status into four groups: liver transplant, malignancy, kidney transplant, and others. The “others” group included patients with chronic immunosuppressive therapy for autoimmune diseases (e.g., systemic lupus erythematosus or rheumatoid arthritis), primary immunodeficiency disorders, and patients on chronic dialysis therapy without solid organ transplantation. Each patient was classified into a single immunosuppression category without overlap. No patients had concurrent immunosuppressive conditions such as simultaneous organ transplantation or active malignancy. Categorization was based on the primary immunosuppressive diagnosis at the time of emergency department admission.

### 2.3. Blood Analysis Measurements

The serum amyloid A (SAA), procalcitonin (PCT), interleukin-6 (IL-6), high-sensitivity C-reactive protein (hs-CRP), and lactate levels were measured using standard commercial assays on automated analyzers (The Human BDNF ELISA kit-Elabscience Biotechnology Co., Ltd., Wuhan, China; Roche Cobas e600-Roche Diagnostics, Mannheim, Germany; Siemens Dade Behring Nephelometer 100-Siemens Healthcare Diagnostics, Marburg, Germany; and Abbott Architect C16000-Abbott Laboratories, Abbott Park, IL, USA, respectively) according to the manufacturers’ protocols. All biochemical assays were performed on automated analyzers that underwent daily internal quality control (QC) checks and periodic external calibration according to the manufacturer specifications. Detailed laboratory procedures are provided in the Supplementary Materials.

### 2.4. Statistical Analysis

Quantitative data were expressed as the mean ± standard deviation for normally distributed variables and as the median (minimum–maximum) for non-normally distributed variables, while categorical data were presented as counts and percentages. The normality of the data distribution was assessed using the Kolmogorov–Smirnov test, and the homogeneity of variances was evaluated with the Levene test. For data analysis, the independent samples *t*-test, Kruskal–Wallis H test, and Mann–Whitney U test were employed where appropriate, based on exact approaches. A chi-square (χ^2^) test or Fisher’s exact test was used for comparison of the categorical variables, depending on the expected frequencies. Post hoc pairwise comparisons following a significant Kruskal–Wallis H test were performed using the Bonferroni-adjusted Mann–Whitney U test. Data analysis was conducted using IBM SPSS Statistics version 29.0 for Windows 11 [18], and a *p* value of <0.05 was considered statistically significant.

## 3. Results

Among the 80 adult patients included in the study, 44 (55%) were female, and 36 (45%) were male. The patients’ ages ranged from 18 to 82 years, with a mean age of 58.50 ± 14.72 years. The results of the patients’ admission blood and urine tests and vital signs are provided in Supplementary Table S1. The body temperatures of the patients ranged from a minimum of 36 °C to a maximum of 40 °C, with a mean value of 37.5 ± 0.8792 °C (Supplementary Figure S1). The minimum systolic blood pressure was 55 mmHg, and the maximum was 179 mmHg, with a mean value of 119.3 ± 22.98 mmHg. The minimum diastolic blood pressure was 27 mmHg, and the maximum was 105 mmHg, with a mean value of 72.5 ± 14.77 mmHg. The minimum oxygen saturation was 75%, and the maximum was 99%, with a mean value of 94.91 ± 4.24%.

### 3.1. Comparison of Blood, Urinalysis Tests, and Vital Signs Between Febrile and Afebrile Patients

The patients were divided into two groups based on their body temperatures: those with a fever (≥37.5 °C) and those without a fever (<37.5 °C). The primary hypothesis evaluated the relationship between fever status (febrile versus afebrile) and clinical variables, including the presenting complaints at ED admission, final diagnosis, patient outcomes, and mortality. No significant differences were observed between the febrile and afebrile patients regarding immunosuppression type, presenting complaints, final diagnosis, or mortality. However, borderline significance was observed between fever status and patient outcomes (e.g., admission to the ward or ICU) (Table 1). In laboratory analyses, the febrile patients exhibited significantly higher admission levels of LDH, IL-6, and PCT compared with the afebrile patients, while the CRP levels did not significantly differ between groups (Table 2). These findings suggest that relying solely on CRP in afebrile patients may not be sufficiently reliable. Additionally, the febrile group had longer hospital stays, indicating greater clinical complexity and resource utilization (Table 2).

### 3.2. Laboratory Markers and Relationship with Mortality

The relationship between the laboratory blood and urinary test results, vital signs, and mortality was examined in the immunosuppressed patients presenting to the emergency department. Low levels of albumin, oxygen saturation (O_2_), platelet count (PLT), and total protein, as well as high levels of lipase and procalcitonin (PCT), were found to be significantly associated with mortality (Table 3). While PCT levels were significantly associated with mortality, the CRP and IL-6 levels did not differ significantly, suggesting that PCT may have superior prognostic value in this context.

### 3.3. Intensive Care Admission and Clinical Parameters

The relationship between intensive care unit (ICU) admissions and the blood and urine test results, as well as the vital signs of the immunosuppressed patients, was analyzed. It was found that low levels of albumin, total protein, and systolic blood pressure, as well as elevated levels of BUN, LDH, NE, and PCT, were significantly associated with ICU admission (Supplementary Table S2). Again, while PCT levels were significantly associated with ICU admission, the CRP and IL-6 levels did not differ significantly, suggesting that PCT may have superior prognostic value in this context.

### 3.4. Clinical Parameters by Immunosuppression Subgroup

The laboratory and urinalysis results, vital signs, and hospitalization durations of the patients were analyzed and statistically compared according to their immunosuppression subgroups (Supplementary Table S3). Clinical and laboratory parameters varied across the immunosuppression subgroups among patients presenting to the emergency department. Liver transplant recipients exhibited significantly higher albumin and ALT levels compared with other groups, suggesting better preserved liver synthetic function. Conversely, the kidney transplant patients demonstrated lower ALT and AST levels, alongside significantly elevated BUN and creatinine levels, reflecting impaired renal function. The patients with malignancy had lower oxygen saturation and systolic blood pressure levels compared with the transplant recipients, indicating a more compromised cardiopulmonary status. Differences in the amylase and lipase levels were also observed across the groups, potentially reflecting underlying organ-specific stress or dysfunction. These findings highlight that different types of immunosuppression are associated with distinct patterns of organ function and clinical severity in the emergency setting.

### 3.5. Clinical Parameters by Final Diagnosis

Immunosuppressed patients presenting to the emergency department were categorized based on their final diagnoses into groups, including cholangitis, urinary tract infection, sepsis, other abdominal infections, respiratory tract infection, and others. The blood test results, length of hospital stay, vital signs, and urinalysis findings of these patients were analyzed and statistically compared (Supplementary Table S4).

Patients diagnosed with cholangitis exhibited higher liver function markers (ALP, ALT, and AST) and higher total protein and direct bilirubin levels compared with other groups, suggesting more prominent hepatobiliary involvement. Conversely, the patients with sepsis demonstrated elevated lactate levels, consistent with systemic hypoperfusion and metabolic stress. The urinary tract infection patients had higher urinalysis WBC counts and nitrite positivity compared with the other groups, reflecting active urinary infections. Additionally, oxygen saturation was higher in the UTI patients compared with those with sepsis or respiratory infections, indicating better preserved cardiopulmonary function. Hospital stay durations also differed significantly; patients with cholangitis and UTIs had longer hospitalizations compared with those with respiratory infections, highlighting the greater clinical complexity and resource needs in these groups. Detailed statistical comparisons are presented in Supplementary Table S4.

### 3.6. Infection Patterns According to Final Diagnosis

Analysis of the presenting complaints and final diagnoses revealed distinct patterns among the immunosuppressed patients presenting to the emergency department (Supplementary Table S5). Patients presenting with shortness of breath were predominantly diagnosed with respiratory tract infections, while those with urinary symptoms or chills were more likely to be diagnosed with urinary tract infections. General condition deterioration was frequently associated with sepsis, and abdominal pain was commonly linked to cholangitis.

Similarly, when evaluating the immunosuppression status, liver transplant recipients were more frequently diagnosed with cholangitis, kidney transplant patients were more frequently diagnosed with urinary tract infections, malignancy patients were more frequently diagnosed with respiratory tract infections, and patients with other immunosuppressive conditions were more frequently diagnosed with sepsis. These findings highlight the strong association between the underlying immunosuppression type, initial clinical presentation, and ultimate diagnosis in this vulnerable population (Supplementary Table S5).

## 4. Discussion

This study highlights the necessity of promptly and thoroughly evaluating immunosuppressed patients in the emergency department (ED), even in the absence of fever. Immunosuppressed individuals often exhibit atypical disease presentations, which can lead to diagnostic delays. Our primary hypothesis explored the relationship between fever status and various clinical variables, including ED admission complaints, final diagnosis, patient outcomes, and mortality. The analysis did not reveal statistically significant differences between the febrile and afebrile immunosuppressed patients across these clinical outcomes. This result reinforces the idea that afebrile presentations may carry similar clinical severity and should not be underestimated. These findings are in line with recent studies that highlight the diagnostic challenges in immunosuppressed patients presenting to the ED, particularly the limited sensitivity of fever in detecting serious infections.

Several investigations have emphasized the value of early biomarker integration, such as PCT, CRP, IL-6, LDH, and albumin, for improving triage and prognostic accuracy in this population [9,16,19,20]. Our study has added value to some of these markers, even in afebrile presentations, by linking them to ICU admission and mortality outcomes.

A borderline association was observed between fever status and patient outcomes, particularly regarding hospital admission, suggesting a possible link between fever and disease severity. Previous studies have reported that febrile immunosuppressed patients tend to experience more complicated disease courses and prolonged hospital stays [21,22]. Our results underscore the need for further investigation with larger cohorts to determine the clinical significance of fever in immunosuppressed patients.

Beyond fever status, our study identified several laboratory markers associated with adverse outcomes. Notably, PCT was significantly associated with both ICU admission and mortality, highlighting its prognostic relevance. In contrast, CRP and IL-6 levels did not differ significantly between the ICU and non-ICU patients, suggesting a more limited utility in this context. Additionally, prolonged hospital stays were observed in patients with elevated admission levels of inflammatory markers, including CRP and PCT, indicating a possible role for these biomarkers in assessing disease burden. Other factors associated with ICU admission included low levels of albumin, total protein, and systolic blood pressure, along with elevated BUN, LDH, and neutrophil levels. Similarly, mortality was linked to low albumin, oxygen saturation, and total protein levels and platelet counts, as well as elevated PCT and lipase levels. These findings underscore the potential of PCT as a particularly useful biomarker for early risk stratification in immunosuppressed patients. These findings suggest that PCT may serve as a valuable prognostic tool, particularly in cases where fever is absent or non-specific [12,17].

The variation in diagnoses based on the underlying immunosuppressive condition further supports the need for a tailored diagnostic approach. Liver transplant recipients were primarily diagnosed with cholangitis, cancer patients commonly presented with respiratory infections, and kidney transplant recipients frequently had urinary tract infections. This highlights the distinct vulnerabilities of each subgroup and the necessity of personalized management strategies.

As a result, the weak association between fever status and clinical outcomes suggests that a more comprehensive evaluation is required for immunosuppressed patients. Future research should focus on refining diagnostic algorithms, integrating additional prognostic biomarkers, and leveraging artificial intelligence tools to enhance risk stratification and clinical decision making.

## 5. Limitations

- The relatively small sample size and the absence of a formal power analysis may limit the generalizability of the findings. However, this study was designed as an exploratory investigation to generate hypotheses and highlight potential biomarker-based diagnostic differences in a real-world ED setting.
- The lack of a control group of immunocompetent patients limits the ability to contextualize biomarker specificity and interpret the primary findings comparatively. However, this study intentionally focused on variations within the immunosuppressed population, a group that remains underexplored in the literature. Future studies incorporating appropriate control groups could further clarify the specificity of the biomarkers assessed.
- Additionally, detailed data on immunosuppressive regimens (e.g., drug types and durations) and standardized severity scores (such as SOFA or qSOFA) were not available at the time of data collection. This reflects common challenges in real-world emergency settings, where rapid clinical decisions often preclude comprehensive scoring.
- Due to the limited sample size, multivariate analysis (e.g., logistic regression) could not be reliably performed without risking overfitting. Future studies with larger cohorts should address this using an appropriate adjustment model.
- Due to the small sample sizes in some subgroups, the results may be sensitive to small variations, and findings should be interpreted as exploratory.

## 6. Conclusions

This study demonstrated that fever status alone is not a reliable predictor of final diagnosis, mortality, or clinical outcomes in immunosuppressed patients. The findings emphasize the importance of incorporating inflammatory biomarkers and a thorough clinical evaluation into the management of these patients. These findings suggest that relying on PCT rather than fever status alone may facilitate earlier identification of critical illness in immunosuppressed patients presenting to the emergency department. Incorporating PCT into clinical pathways may improve outcomes by enabling timely intervention.

Our results contribute to the growing body of literature on managing immunosuppressed patients in the emergency department by providing evidence-based insights that can improve care for this vulnerable population. Future studies should aim to validate these findings in larger cohorts, refine diagnostic and therapeutic strategies, and develop structured diagnostic pathways specifically tailored to immunosuppressed patients in the emergency department setting to enhance clinical decision making and optimize patient outcomes.

## Figures and Tables

**Table 1:** Comparison of febrile and afebrile groups based on immunosuppression status, complaints at ED admission, final diagnosis, outcome, and mortality.

Chi-Square Analyses		Fever Status		$p\;\mathrm{Value}\;(\chi^{2})$
		Afebrile	Febrile	
		N	N	
Immunosuppression status	Liver transplantation	11	14	0.839 (0.845)
	Kidney transplantation	14	12	
	Malignancy	6	6	
	Others	7	10	
Complaint at ED admission	Shortness of breath	10	10	0.801 (1.645)
	Burning in urine, chills, shivering	4	8	
	General condition disorder, weakness	8	8	
	Abdominal pain, fever	8	10	
	Nausea, vomiting, other	8	6	
Final diagnosis	Cholangitis	4	10	0.656 (3.286)
	Urinary tract infection	6	8	
	Sepsis	6	6	
	Other abdominal infections	6	5	
	Respiratory tract infection	10	7	
	Others	6	6	
Outcome	Hospitalization	35	32	0.054 (3.713)
	ICU	3	10	
Mortality	No	35	38	0.797 (0.066)
	Yes	3	4	

**Table 2:** Clinical and laboratory parameters of febrile and afebrile immunosuppressed patients.

Parameter	Afebrile	Febrile	*p* Value
	Med [Min–Max]	Med [Min–Max]	
**Fever (°C)**	**36.8 [36–37.3]**	**38.05 [37.5–40]**	**0.001**
SBP (mmHg)	118.5 [56–161]	117.5 [55–179]	0.765
DBP (mmHg)	72.5 [33–98]	75 [27–105]	0.693
O_2_ (%)	96 [84–99]	96 [75–99]	0.265
WBC (10^3^/µL)	7.49 [0.36–19.8]	8.73 [0.43–223.1]	0.449
HB (gr/dL)	10.25 [6.2–15.7]	10.85 [6.6–15]	0.693
HCT (%)	31.05 [20.1–47.5]	32.25 [9.5–45.8]	0.658
LY No. (10^3^/µL)	1.005 [0.19–3.98]	0.87 [0.05–209.84]	0.965
MPV (fL)	10.55 [8.5–13.9]	10.5 [8.2–106]	0.776
NE No. (10^3^/µL)	5.06 [0–18.87]	6.775 [0.08–34.86]	0.851
PLT (10^3^/µL)	203 [6–726]	185 [11–491]	0.547
Glucose (mg/dL)	125.5 [87–624]	123.5 [62–331]	0.205
Creatinine (mg/dL)	1.11 [0.58–8]	1.04 [0.37–8.4]	0.633
BUN (mg/dL)	23.735 [6.42–85.92]	19.83 [4.81–83.69]	0.430
AST (U/L)	26.5 [7–528]	39.5 [8–1880]	0.061
ALT (U/L)	23.5 [6–414]	27 [6–338]	0.488
ALP (U/L)	117.5 [19–1107]	183 [44–1428]	0.075
GGT (U/L)	57 [12–1402]	96 [10–1846]	0.119
**LDH (U/L)**	**247.5 [139–1092]**	**300.5 [141–3543]**	**0.050**
Total bilirubin (g/dL)	0.845 [0.23–30.84]	0.995 [0.27–13.92]	0.806
Direct bilirubin (g/dL)	0.405 [0.13–21.39]	0.515 [0.17–8.81]	0.576
Albumin (g/dL)	2.9 [1.7–4.1]	2.95 [1.7–3.9]	0.563
Total protein (g/dL)	6.95 [5.1–8.7]	6.65 [3.7–8.4]	0.143
Amylase (U/L)	52 [9–1080]	53 [4–123]	0.769
Lipase (U/L)	21.5 [4–125]	27.5 [4–142]	0.491
Sodium (mmol/L)	135 [128–142]	135 [120–143]	0.753
Potassium (mmol/L)	4.305 [3.41–6.23]	4.26 [2.59–5.7]	0.441
Ph	7.395 [7.3–7.65]	7.39 [0–7.54]	0.870
Lactate (mmol/L)	12.4 [4.4–58.43]	14 [5.6–76]	0.479
**IL6 (pg/mL)**	**75.755 [2.12–5000]**	**111.55 [1.5–5000]**	**0.006**
CRP (mg/dL)	6.8 [0.311–39.2]	10.15 [0.382–30.1]	0.092
**PCT (ng/mL)**	**0.36 [0.028–100]**	**1.79 [0.071–100]**	**0.006**
SAA (ng/mL)	33.627 [0.12–77.03]	32.785 [0.38–71.26]	0.939
UA nitrite	1 [1–2]	1 [1–2]	0.595
UA RBCs	0.5 [0–728]	1 [0–87]	0.930
UA WBCs	1 [0–386]	2 [0–562]	0.303
**Hospital stay (days)**	**6 [1–91]**	**10.5 [1–56]**	**0.012**

ALP = alkaline phosphatase; ALT = alanine aminotransferase; AST = aspartate aminotransferase; BUN = blood urea nitrogen; CRP = C-reactive protein; DBP = diastolic blood pressure; GGT = gamma-glutamyl transferase; HB = hemoglobin; HCT = hematocrit; IL-6 = interleukin 6; LDH = lactate dehydrogenase; LY = lymphocyte; MPV = mean platelet volume; NE = neutrophil; O_2_ = oxygen saturation; PCT = procalcitonin; pH = potential of hydrogen; PLT = platelet; SAA = serum amyloid A; SBP = systolic blood pressure; UA RBCs = urinalysis, red blood cells; UA WBCs = urinalysis, white blood cells; WBCs = white blood cells.

**Table 3:** The relationship between the blood and urine tests, vital signs, and mortality in immunosuppressed patients.

	Mortality (-)	Mortality (+)	
Parameter	Med [Min–Max]	Med [Min–Max]	*p* Value
Fever (°C)	37.5 [36.0–40.0]	37.5 [36.8–40.0]	0.679
SBP (mmHg)	119 [56–179]	107 [55–138]	0.122
DBP (mmHg)	75 [33–105]	63 [27–100]	0.167
**O_2_ (%)**	**96 [81–99]**	**92 [75–96]**	**0.001**
WBC (10^3^/µL)	8.54 [0.36–223.1]	7.92 [0.49–14.29]	0.616
HB (gr/dL)	10.5 [ 6.2–15.7]	9.9 [7.6–13.4]	0.312
HCT (%)	32.9 [9.5–47.5]	27.6 [21.9–43.7]	0.258
LY# (10^3^/µL)	0.99 [0.11–209.84]	0.87 [0.05–3.01]	0.891
MPV (fL)	10.40 [8.20–31.10]	11.00 [8.80–106]	0.501
NE# (10^3^/µL)	5.74 [0.00–34.86]	4.47 [0.27–12.30]	0.603
**PLT (10^3^/µL)**	**203 [11–726]**	**102 [6–354]**	**0.021**
Glucose (mg/dL)	124 [62–624]	123 [83–308]	0.680
Creatinine (mg/dL)	1.05 [0.49–8.40]	2.41 [0.37–5.18]	0.110
BUN (mg/dL)	20.76 [4.81–85.92]	25.10 [17.22–76.32]	0.089
AST (U/L)	32 [7–1880]	23 [14–209]	0.755
ALT (U/L)	24 [6–414]	27 [13–140]	0.957
ALP (U/L)	129 [19–1428]	185 [65–826]	0.506
GGT (U/L)	83 [10–1846]	123 [38–511]	0.491
LDH (U/L)	265 [139–3543]	263 [144–995]	0.590
Total bilirubin (g/dL)	0.94 [0.23–21.71]	2.25 [0.27–30.84]	0.238
Direct bilirubin (g/dL)	0.43 [0.13–13.20]	1.53 [0.17–21.39]	0.152
**Albumin (g/dL)**	**3.0 [1.7–4.1]**	**2.2 [1.9–3.3]**	**0.015**
**Total protein (g/dL)**	**6.9 [3.7–8.7]**	**5.7 [5.1–8.1]**	**0.039**
Amylase (U/L)	53 [4–1080]	66 [27–161]	0.444
**Lipase (U/L)**	**22 [4–142]**	**38 [18–81]**	**0.034**
Sodium (mmol/L)	135 [120–143]	135 [125–141]	0.767
Potassium (mmol/L)	4.28 [2.59–6.23]	4.32 [2.70–5.59]	0.812
pH	7.39 [7.28–7.65]	7.38 [6.99–7.49]	0.454
Lactate (mmol/L)	12.90 [4.40–58.43]	14.10 [9.60–76]	0.135
IL6 (pg/mL)	97.04 [1.50–5000]	193.10 [70.6–5000]	0.079
CRP (mg/dL)	7.880 [0.31–39.2]	11.200 [0.74–30.1]	0.251
**PCT (ng/mL)**	**0.580 [0.02–100]**	**3.190 [0.72–100]**	**0.021**
SAA (ng/mL)	31.980 [0.12–77.03]	44.860 [13.13–51.98]	0.677
UA nitrite	1 [1–2]	1 [1–1]	0.397
UA RBCs	1 [0–728]	0 [0 –1]	0.122
UA WBCs	1 [0–562]	0 [0–5]	0.177
Hospital stay (days)	7 [1–91]	4 [1–47]	0.902

ALP = alkaline phosphatase; ALT = alanine aminotransferase; AST = aspartate aminotransferase; BUN = blood urea nitrogen; CRP = C-reactive protein; DBP = diastolic blood pressure; GGT = gamma-glutamyl transferase; HB = hemoglobin; HCT = hematocrit; IL-6 = interleukin 6; LDH = lactate dehydrogenase; LY = lymphocyte; MPV = mean platelet volume; NE = neutrophil; O_2_ = oxygen saturation; PCT = procalcitonin; pH = potential of hydrogen; PLT = platelet; SAA = serum amyloid A; SBP = systolic blood pressure; UA nitrite = urinalysis nitrite; UA RBCs = urinalysis, red blood cells; UA WBCs = urinalysis, white blood cells; WBC = white blood cells.

## Data Availability

The raw data supporting the conclusions of this article will be made available by the authors on request.

## Supplementary Materials

The following supporting information can be downloaded at https://www.mdpi.com/article/10.3390/medicina61050889/s1. Table S1: Blood and urinary test results and vital signs for all immunosuppressed patients; Table S2: The relationship between blood and urine tests, vital signs, and admission to hospitalization and ICU in immunosuppressed patients; Table S3: Statistical analyses of the distribution of blood, urine tests, and vital signs according to immunosuppression status; Table S4: Clinical and Laboratory Parameters of Immunosuppressed Patients with Fever Based on Final Diagnosis; Table S5: Percentage* of final diagnoses within the main complaint in immunosuppressed patients presenting; Figure S1: Fever distribution of all immunosuppressed patients included in the study.

## Abbreviations

The following abbreviations are used in this manuscript:

ALP	Alkaline phosphatase
ALT	Alanine aminotransferase
AST	Aspartate aminotransferase
BUN	Blood urea nitrogen
CRP	C-reactive protein
ED	Emergency department
ICU	Intensive care unit
LDH	Lactate dehydrogenase
NE	Neutrophil
PCT	Procalcitonin
PLT	Platelet count
RBC	Red blood cell
UA WBC	Urinary white blood cell count
WBC	White blood cell
