# Supplementary material for: Prospective Evaluation of Clinical and Laboratory Profiles of Febrile and Afebrile Immunosuppressed Patients Presenting to the Emergency Department
Source: Medicina (Kaunas). 2025 May 14;61(5):889. doi: 10.3390/medicina61050889 (PMC12112846; doi:10.3390/medicina61050889)
Supplement: Supplementary file 1 [file medicina-61-00889-s001.zip › medicina-3565194-supplementary.pdf]

Supplementary File

Prospective Evaluation of Clinical and Laboratory Profiles of Febrile and Afebrile Immunosuppressed Patients Presenting to the Emergency Department

Tuğrul Topal [1], Esra Pamukçu [2,*], Muhammet Gökhan Turtay [3], Gülşen Yalçın [4], Harun Kürşat Şahingil [5] and Mehmet Sezer [6]

[1] Emergency Medicine Clinic of Necip Fazıl City Hospital, 46050 Kahramanmaraş, Turkey;
[2] Department of Statistics, Faculty of Science, Firat University, 23119 Elazığ, Turkey
[3] Emergency Medicine Clinic of Konya City Hospital, 42020 Konya, Turkey
[4] Department of Medical Biochemistry, Turgut Ozal Medical Center, Inonu University, 44210 Malatya, Turkey
[5] Emergency Medicine Clinic of Yeşilyurt Hasan Çalık State Hospital, 44920 Malatya, Turkey
[6] Emergency Medicine Clinic of Besni State Hospital, 02300 Adıyaman, Turkey
* Correspondence: epamukcu@firat.edu.tr

METHODS OF BLOOD ANALYSIS MEASUREMENT

- Measurement of Serum Amyloid A Levels: Blood samples were collected from patients into plain biochemical tubes and centrifuged at 3500-4000 rpm in a laboratory centrifuge. The serum was separated into polypropylene tubes and stored at -80°C until the day of the analysis. On the analysis day, the frozen samples were thawed at room temperature and vortexed before analysis to ensure homogenization.

- Serum Amyloid A levels were measured using a commercial enzyme-linked immunosorbent assay (ELISA) kit (Elabscience Biotechnology Co., Ltd., China – Human BDNF ELISA Kit) and analyzed with a Biotek Synergy H1 model device. In accordance with the procedure, 100 μL of standard or sample was pipetted into the antibody-coated microplate wells and incubated at 37°C for 90 minutes. During the incubation period, Serum Amyloid A in the sample bound to the immobilized antibodies in the wells. After incubation, the wells were emptied and 100 μL of biotinylated detection antibody was added to each well, followed by a 60-minute incubation at 37°C. The microplate wells were washed three times with washing solution. Then, 100 μL of streptavidin-HRP was added to each well and incubated for 30 minutes at 37°C. After incubation, the wells were washed five times with washing solution, and 90 μL of TMB substrate was added, followed by a 15-minute incubation in the dark at 37°C. Afterward, 50 μL of stop solution was added to terminate the reaction. The optical density of the samples was read at 450 nm using a microplate reader. The concentrations of the samples were calculated based on a standard curve and reported in ng/mL. The kit's measurement range was 1.25-80 ng/mL, with an intra-assay CV of <6%.

- Measurement of PCT Levels: PCT levels were measured using the Roche Cobas e600 model device with the electrochemiluminescence method (Roche Diagnostics GmbH, D-68298 Mannheim, Germany). The results were evaluated in ng/mL using a fully automated device that underwent control and calibration. The measurement range was defined as 0.02-100 ng/mL.

- Measurement of IL-6 Levels: IL-6 levels were measured using the Roche Cobas e600 model device with the electrochemiluminescence method (Roche Diagnostics GmbH, D-68298 Mannheim, Germany). The results were evaluated in pg/mL using a fully automated device that underwent control and calibration. The measurement range was defined as 1.5-5000 pg/mL.

- Measurement of hs-CRP Levels: Serum hs-CRP levels were measured using the nephelometric method with the Siemens Dade Behring Nephelometer 100 model analyzer (Siemens Healthcare Diagnostic Products GmbH, Emil-von-Behring-Str. 76, 35041 Marburg, Germany). Values below 0.33 mg/L were considered normal according to the hs-CRP results obtained.

- Measurement of Lactate Levels: Serum lactate levels were measured using the spectrophotometric method with the Abbott C16000 model auto-analyzer. The samples were analyzed within 30 minutes after calibration and control of the device.

Supplementary Table S1: Blood and urinary test results and vital signs for all immunosuppressed patients

Parameter	Mean±Std. Deviation	Min	Max
Fever (°C)	37,5±0,88	36	40
SBP (mmHg)	119,3±22,98	55	179
DBP (mmHg)	72,5±14,77	27	105
O_2 (%)	94,91±4,25	75	99
WBC (10^3/Ul)	11,81±24,87	0,36	223,1
HB (gr/dL)	10,7±2,23	6,2	15,7
HCT (%)	32,09±7,29	9,5	47,5
MPV (fL)	12,03±10,94	8,2	106
LY# (10^3/uL)	3,79±23,34	0,05	209,84
NE# (10^3/uL)	6,79±6,01	0	34,86
PLT (10^3/uL)	219,7±141,52	6	726
Glucose(mg/dL)	142,69±75,26	62	624
Creatinine (mg/dL)	1,76±1,73	0,37	8,4
BUN (mg/dL)	27,7983±19,69	4,81	85,92
AST (U/L)	87,05±220,76	7	1880
ALT (U/L)	61,01±84,32	6	414
ALP (U/L)	245,7±266,02	19	1428
GGT (U/L)	225,96±331,29	10	1846
LDH (U/L)	408,83±537,75	139	5343
Total bilirubin(g/dL)	2,74±4,95	0,23	30,84
Direct bilirubin(g/dL)	1,66±3,25	0,13	21,39
Albumin (g/dL)	2,91±0,62	1,7	4,1
Total protein (g/dL)	6,72±0,92	3,7	8,7
Amylase (U/L)	67,85±118,76	4	1080
Lipase (U/L)	30,7±27,98	4	142
Sodium (mmol/L)	134,59±4,72	120	143
Potassium (mmol/L)	4,3±0,7	2,59	6,23
pH	7,31±0,83	6,99	7,65
Lactate (mmol/L)	17,02±13,14	4,4	76
IL6 (pg/mL)	562,74±1330,55	1,5	5000
CRP (mg/dL)	10,37±8,11	0,31	39,2
PCT (ng/mL)	10,94±25,9	0,02	100
SAA (ng/mL)	33,7±21,18	0,12	77,03
UA Nitrite	1,09±0,28	1	2
UA WBC	38,31±105,94	0	562
UA RBC	12,81±81,78	0	728
Hospital Stay (day)	11,77±14,13	1	91

ALP: Alkaline Phosphatase; ALT: Alanine Aminotransferase; AST: Aspartate Aminotransferase; BUN: Blood Urea Nitrogen; CRP: C-Reactive Protein; DBP: Diastolic Blood Pressure; GGT: Gamma-Glutamyl Transferase; HB: Hemoglobin; HCT: Hematocrit; IL-6: Interleukin 6; LDH: Lactate Dehydrogenase; LY: Lymphocyte; MPV: Mean Platelet Volume; NE: Neutrophil; O_2: Oxygen Saturation; PCT: Procalcitonin; pH: Potential of Hydrogen; PLT: Platelet; SAA: Serum Amyloid A; SBP: Systolic Blood Pressure; UA Nitrite: Urinalysis Nitrite; UA RBC: Urinalysis Red Blood Cell; UA WBC: Urinalysis White Blood Cell; WBC: White Blood Cell.

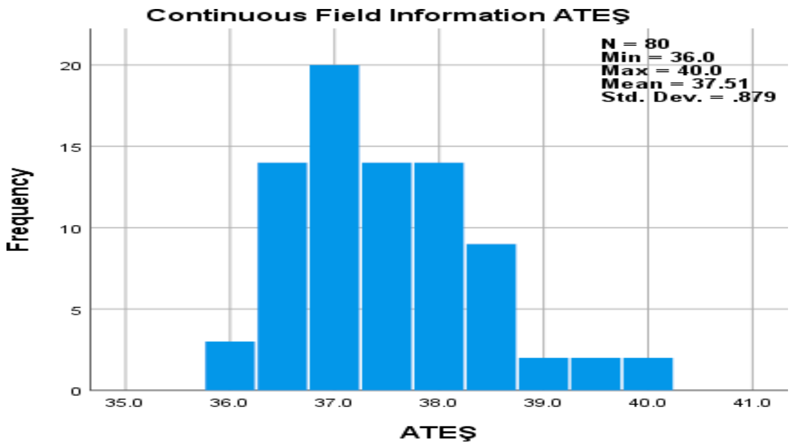

Supplementary Figure S1: Fever distribution of all immunosuppressed patients included in the study

Statistical tests used: Independent-samples t-test for normally distributed continuous variables, Mann–Whitney U test for non-normally distributed continuous variables, Chi-square test for categorical variables, and Fisher's exact test when expected cell counts were less than 5.

Supplementary Table S2: The relationship between blood and urine tests, vital signs, and admission to hospitalization and ICU in immunosuppressed patients.

	Outcome		
	Hospitalization	ICU	
Parameter	Med[min- max]	Med[min- max]	**p-value**
Fever (°C)	37.2 [36.2- 40.0]	37.6 [36.0- 40.0]	0.357
**SBP (mmHg)**	**121 [56- 179]**	**110 [55- 154]**	**0.028**
DBP (mmHg)	75 [33- 105]	65 [27- 100]	0.199
O_2 (%)	96 [81- 99]	95 [75- 98]	0.075
WBC (10^3/Ul)	7.82 [0.36- 223.10]	10.77 [0.49- 38.83]	0.119
HB (gr/dL)	11.1 [6.6- 15.7]	9.9 [6.2- 15.0]	0.267
HCT (%)	32.9 [9.5- 47.5]	28.9 [20.1- 45.8]	0.273
LY# (10^3/uL)	0.94 [0.11- 209.84]	1.18 [0.05- 3.01]	0.705
MPV (fL)	10.60 [8.20- 31.10]	10.40 [8.90- 106.00]	0.566
**NE# (10^3/uL)**	**5.16 [0.00- 18.87]**	**9.07 [0.38- 34.86]**	**0.046**
PLT (10^3/uL)	189 [6- 726]	269 [32- 491]	0.499
Glucose (mg/dL)	132 [79- 624]	118 [62- 308]	0.186
Creatinine (mg/dL)	1.05 [0.49- 8.40]	1.67 [0.37- 5.18]	0.422
**BUN (mg/dL)**	**20.38 [4.81- 85.92]**	**27.81 [6-28- 83.69]**	**0.040**
AST (U/L)	30 [7- 528]	65 [12- 1880]	0.113
ALT (U/L)	24 [6- 414]	32 [6- 183]	0.502
ALP (U/L)	124 [19- 1428]	201 [41- 985]	0.083
GGT (U/L)	83 [10- 1846]	175 [15- 918]	0.240
**LDH (U/L)**	**252 [139- 3499]**	**490 [159- 3543]**	**0.010**
Total Bilirubin (g/dL)	0.93 [0.23- 21.71]	1.42 [0.23- 30.84]	0.639
Direct Bilirubin (g/dL)	0.43 [0.13- 13.20]	0.94 [0.15- 21.39]	0.506
**Albumin (g/dL)**	**3.1 [1.7- 4.1]**	**2.5 [1.7- 3.3]**	**0.002**
**Total protein (g/dL)**	**6.9 [5.1- 8.7]**	**6.3 [3.7- 8.4]**	**0.018**
Amylase (U/L)	51 [9- 1080]	55 [4- 161]	0.705
Lipase (U/L)	21 [4- 142]	41 [4- 81]	0.064
Sodium (mmol/L)	135 [125-143]	135 [120- 141]	0.578
Potassium (mmol/L)	4.25 [2.59- 6.23]	4.42 [2.70- 5.75]	0.230
pH	7.40 [7.31- 7.65]	7.39 [0.00- 7.52]	0.264
Lactate (mmol/L)	12.60 [4.40- 58.43]	17.70 [5.60- 76.00]	0.150
IL6 (pg/mL)	99.23 [1.50- 5000.00]	88.15 [4.92- 5000.00]	0.438
CRP (mg/dL)	8.61 [0.31- 39.20]	7.77 [0.51- 30.10]	0.995
**PCT (ng/mL)**	**0.57 [0.02- 100.00]**	**3.19 [0.21- 100.00]**	**0.009**
SAA (ng/mL)	31.98 [0.12- 77.03]	44.86 [0.38- 71.26]	0.700
UA Nitrite	1[1-2]	1[1-1]	0,225
UA RBC	1 [0- 728]	0 [0- 36]	0.290
UA WBC	1 [0- 562]	1 [0- 370]	0.770
Hospital stay duration (day)	7 [1- 51]	14 [1-91]	0.207

ALP: Alkaline Phosphatase; ALT: Alanine Aminotransferase; AST: Aspartate Aminotransferase; BUN: Blood Urea Nitrogen; CRP: C-Reactive Protein; DBP: Diastolic Blood Pressure; GGT: Gamma-Glutamyl Transferase; HB: Hemoglobin; HCT: Hematocrit; IL-6: Interleukin 6; LDH: Lactate Dehydrogenase; LY: Lymphocyte; MPV: Mean Platelet Volume; NE: Neutrophil; O_2: Oxygen Saturation; PCT: Procalcitonin; pH: Potential of Hydrogen; PLT: Platelet; SAA: Serum Amyloid A; SBP: Systolic Blood Pressure; UA Nitrite: Urinalysis Nitrite; UA RBC: Urinalysis Red Blood Cell; UA WBC: Urinalysis White Blood Cell; WBC: White Blood Cell.

Supplementary Table S3: Statistical analyses of the distribution of blood, urine tests, and vital signs according to immunosuppression status

Parameter	Immunosuppression status				p-value
	Liver transplantation	Malignancy	Kidney transplantation	Others	
	Med[min-max]	Med[min-max]	Med[min- max]	Med[min-max]	
Fever (°C)	37.5[36.5-38.8]	37.2[36.0-40.0]	37.8[36.4-39.3]	37.5[36.2-40.0]	0.760
**SBP (mmHg)**	**126[a][93-157]**	111[56-172]	124[105-147]	116[55-179]	**0.044**
DBP (mmHg)	76[39-93]	64[33-105]	82[59-98]	77[27-100]	0.071
**O_2 (%)**	97[88-99]	**95[c-d][81-99]**	98[84-99]	96[75-98]	**0.015**
WBC (10^3/Ul)	7.82[1.35-38.83]	10.29[0.36-223.1]	5.53[1.28-17.76]	8.73[0.43-19.8]	0.0523
HB (gr/dL)	11.1[8.1-15]	10.3[6.2-15.7]	10.4[7.1-13.6]	10.4[6.6-14.4]	0.616
HCT (%)	33.1[24.6-45.8]	28.7[9.5-47.5]	31.6[21.4-41.8]	30.3[20.9-44.3]	0.493
LY# (10^3/uL)	0.94[0.14-3.18]	1.19[0.19-209.84]	0.94[0.13-1.71]	0.74[0.05-3.98]	0.280
MPV (fL)	10.70[9-12.6]	10.25[8.20-31.10]	10.90[9.20-11.30]	10.60[8.50-106]	0.570
NE# (10^3/uL)	6.48[0.77-34.86]	6.94[0-33.40]	3.67[1.13-14.66]	5.36[0.08-18.87]	0.790
PLT (10^3/uL)	203[12-445]	227[6-726]	195[106-457]	165[21-491]	0.824
Glucose(mg/dL)	132[91-624]	113[62-203]	122[79-150]	129[71-321]	0.746
**Creatinine (mg/dL)**	1.05[0.59-2.15]	0.91[0.37-8.40]	**2.91[1.42-5.54][a-b-c]**	1.13[0.49-6.60]	**0.008**
**BUN (mg/dL)**	20.13[6.19-83.69]	20.55[4.81-67.89]	**46.7[24.69-85.92][a-b-c]**	23.59[6.28-76.32]	**0.033**
**AST (U/L)**	**47[16-528][a]**	22[7-320]	**14[8-18][a-b-c]**	34[14-1880]	**0.001**
**ALT (U/L)**	**57[10-414][a-b]**	22[6-101]	**12[6-22][c-b]**	21[6-306]	**0.0001**
ALP (U/L)	198[60-985]	117[41-1428]	97[53-228]	125[19-842]	0.064
GGT (U/L)	138[16-918]	105[12-1846]	38[16-199]	54[10-1402]	0.086
LDH (U/L)	250[144-576]	325[139-3499]	342[173-604]	240[141-3543]	0.111
Total bilirubin(g/dL)	1.16[0.23-21.71]	1.00[0.23-16.91]	0.67[0.30-0.99]	0.69[0.23-30.84]	0.167
Direct bilirubin(g/dL)	0.63[0.13-13.20]	0.46[0.15-9.23]	0.30[0.14-0.50]	0.35[0.14-21.39]	0.065
**Albumin (g/dL)**	**3.3[1.8-3.9][a-b]**	2.8[1.7-3.9]	3.1 [2.2-4.1]	2.6[1.7-3.9]	**0.024**
Total protein(g/dL)	7.1[5.3-7.9]	6.6[3.7-8.3]	6.0[5.0-7.6]	6.6[5.1-8.7]	0.102
**Amylase (U/L)**	**47[20-114][d]**	**38[4-1080][b-d]**	74[51-163]	63[17-161]	**0.024**
**Lipase (U/L)**	**18[4-68][b-d]**	**19[4-129][b-d]**	43[19-95]	37[6-142]	**0.005**
Sodium (mmol/L)	136[126-142]	135[120-143]	137[125-143]	134[125-142]	0.450
Potassium (mmol/L)	4.18[3.20-5.57]	4.27[2.70-5.75]	4.22[3.77-5.23]	4.31[2.59-6.23]	0.871
**pH**	**7.38[7.31-7.56][a]**	7.41[7.32-7.54]	**7.36[7.31-7.45][a-b]**	7.42[6.99-7.65]	**0.026**
Lactate (mmol/L)	13.90[4.40-58.43]	12.75[5.9-73.40]	14.60[6.50-25.90]	13.40[5.60-76.00]	0.909
IL6 (pg/mL)	44.51[1.50-4765]	92.60[2.12-5000]	257.7[3.77-5000]	122.90[6.87-5000]	0.051
CRP (mg/dL)	6.26[0.38-25.2]	11.7[0.51-25.4]	4.0[0.31-25.3]	9.9[0.57-39.2]	0.079
PCT (ng/mL)	1.02[0.07-100]	0.59[0.03-100]	0.45[0.04-84.24]	0.658[0.10-100]	0.483
SAA (ng/mL)	36.96[0.34-77.03]	30.76[0.29-62.6]	41.94[9.45-60.92]	33.35[0.12-71.26]	0.759
**UA Nitrite**	1[1-1]	1[1-2]	1[1-2]	**1[a-c] [1-2]**	**0.033**
UA RBC	0[0-728]	1[0-87]	1[0-36]	0[0-44]	0,462
UA WBC	1[0-188]	1[0-131]	10[1-447]	3[0-562]	0.106
Hospital stay (day)	8[1-91]	5[1-21]	11[1-51]	8[1-56]	0.129

ALP: Alkaline Phosphatase; ALT: Alanine Aminotransferase; AST: Aspartate Aminotransferase; BUN: Blood Urea Nitrogen; CRP: C-Reactive Protein; DBP: Diastolic Blood Pressure; GGT: Gamma-Glutamyl Transferase; HB: Hemoglobin; HCT: Hematocrit; IL-6: Interleukin 6; LDH: Lactate Dehydrogenase; LY: Lymphocyte; MPV: Mean Platelet Volume; NE: Neutrophil; O_2: Oxygen Saturation; PCT: Procalcitonin; pH: Potential of Hydrogen; PLT: Platelet; SAA: Serum Amyloid A; SBP: Systolic Blood Pressure; UA Nitrite: Urinalysis Nitrite; UA RBC: Urinalysis Red Blood Cell; UA WBC: Urinalysis White Blood Cell; WBC: White Blood Cell.

a: There is a significant difference according to malignancy ($p<0.05$).

b: There is a significant difference according to others ($p<0.05$).

c: There is a significant difference according to liver transplant ($p<0.05$).

d: There is a significant difference according to kidney transplant ($p<0.05$).

Supplementary Table S4: Clinical and Laboratory Parameters of Immunosuppressed Patients with Fever Based on Final Diagnosis

	Final Diagnosis						
	Cholangitis	Urinary tract infection	Sepsis	Other abdominal infections	Respiratory tract infection	Others	
Parameters	Med[Min-Max]	Med[Min-Max]	Med[Min-Max]	Med[Min-Max]	Med[Min-Max]	Med[Min-Max]	p-value
Fever (°C)	37.5[36.5-38.5]	37.6[36.2-38.8]	37.4[36.2-39.1]	37.3[36.5-40]	37[36.3-40]	37.6[36-39.5]	0.907
SBP (mmHg)	124[108-154]	117[95-157]	100[55-165]	112[93-179]	121[87-161]	113[88-172]	0.213
DBP(mmHg)	80[56-89]	76[59-93]	62[27-100]	75[39-92]	71[53-98]	67[55-105]	0.405
**O_2 (%)**	96[88-99]	**98[c,e][96-99]**	95[75-98]	96[95-98]	95[81-98]	95[84-99]	**0.003**
WBC (10^3/Ul)	8.58[1.35-17.16]	11.98[1.28-38.83]	7.4[0.36-34.54]	7.82[4.54-14.98]	7.92[0.7-223.1]	7.49[0.54-26.67]	0.851
**HB (gr/dL)**	**12.8[d.f][8.1-14.9]**	11.6[6.6-15]	10.1[7.2-14]	9.1[7.1-13.4]	11.1[7-15.7]	9.3[6.2-12.9]	**0.047**
**HCT (%)**	36.8[24.6-44]	36[20.9-45.8]	28.7[21.6-43.7]	**27.4[a.b][21.4-39.4]**	33.2[9.5-47.5]	**27.1[a.b.e][20.1-38.8]**	**0.023**
LY# (10^3/uL)	0.87[0.14-4.11]	1.13[0.13-3.18]	0.62[0.05-2.4]	0.94[0.44-1.46]	0.87[0.37-209.84]	1.12[0.23-5.59]	0.594
MPV (fL)	11.25[9.5-12.6]	10.35[9.2-13.4]	10.15[8.9-106]	10.7[9.4-13.1]	10.3[8.5-31.1]	10.05[8.2-11.6]	0.098
NE# (10^3/uL)	6.5[0.77-11.09]	8.2[1.13-34.86]	4.21[0-33.4]	5.74[3.44-13.1]	4.89[0.77-18.87]	4.96[0.03-12.3]	0.43
PLT (10^3/uL)	155[65-429]	305[12-457]	152[32-360]	148[21-491]	201[32-726]	146[6-343]	0.116
Glucose(mg/dL)	130[71-624]	114[79-154]	131[62-308]	129[87-321]	129[83-239]	123[80-203]	0.571
Creatinine (mg/dL)	0.94[0.64-2.15]	1.53[0.59-8]	0.88[0.49-5.18]	1.32[0.66-6.6]	1.11[0.58-4.69]	1.11[0.37-8.4]	0.34
BUN (mg/dL)	17.37[6.19-34.54]	24.88[6.42-83.69]	17.85[7.21-76.32]	30.46[6.28-67.6]	24.05[7.19-67.89]	28.99[4.81-85.92]	0.446
**AST (U/L)**	**105[b.e][17-297]**	22[8-139]	38[11-320]	31[14-1880]	25[11-117]	20[7-528]	**0.009**
**ALT (U/L)**	**125[b.d.e.f][10-414]**	21[8-56]	23[10-140]	24[6-170]	22[6-113]	15[6-401]	**0.001**
**ALP (U/L)**	**314[e][101-803]**	108[54-985]	193[44-1107]	141[53-346]	109[19-826]	121[41-1428]	**0.038**
**GGT (U/L)**	**348[b.e.f][83-1402]**	39[10-918]	89[19-1048]	66[16-286]	89[17-543]	51[12-1846]	**0.002**
LDH (U/L)	252[164-780]	301[186-834]	315[141-1092]	250[144-3543]	262[139-995]	238[144-3499]	0.855
**Total bilirubin(g/dL)**	**2.25[e][0.28-13.92]**	0.72[0.23-2.62]	3.57[0.27-30.84]	0.74[0.23-7.56]	0.63[0.39-2.21]	1.07[0.23-21.71]	**0.008**
**Direct bilirubin (g/dL)**	**1.57[b.e][0.19-8.81]**	0.4[0.13-1.33]	2.11[0.17-21.39]	0.41[0.14-3.23]	0.33[0.2-0.94]	0.54[0.15-13.2]	**0.003**
**Albumin (g/dL)**	**3.3[c.d.f][1.8-3.9]**	**3.3[c.d.f][2.2-3.8]**	2.6[1.7-3.7]	2.6[1.7-3.7]	3.1[2-4.1]	2.7[1.9-3.9]	**0.019**
**Total protein(g/dL)**	**7.2[c.f][6-8.4]**	7[5.9-7.6]	6.3[3.7-8.3]	6.9[5-8.5]	7[5.1-8.7]	6.1[5.1-7.4]	**0.008**
Amylase (U/L)	40[20-80]	55[26-101]	44[4-161]	69[18-99]	57[9-1080]	50[15-123]	0.352
Lipase (U/L)	13[4-68]	35[7-95]	26[4-81]	47[6-125]	22[4-142]	19[4-51]	0.196
Sodium (mmol/L)	135[129-139]	136[125-143]	133[120-141]	135[132-141]	135[125-143]	136[128-142]	0.33
Potassium (mmol/L)	4.17[3.2-4.72]	4.58[3.51-5.23]	4.18[2.59-5.59]	4.32[3.55-6.23]	4.32[3.37-4.78]	3.9[2.7-5.75]	0.516
pH	7.38[7.3-7.45]	7.37[7.28-7.52]	7.4[6.99-7.54]	7.44[7.33-7.65]	7.39[7.34-7.54]	7.4[7.32-7.5]	0.164
**Lactate (mmol/L)**	14.7[4.4-30.3]	11.75[6.3-29]	**21.00[e][5.9-76]**	19.6[5.6-39.5]	9.2[6.5-73.4]	12.35[5.9-58.43]	**0.025**
IL6 (pg/mL)	54.36[1.5-5000]	139.4[12.48-50000]	199.8[36.28-5000]	102.3[5.35-4765]	50.05[2.12-386.1]	66.88[9-3219]	0.064
CRP (mg/dL)	7.995[1.35-19.3]	7.42[1.29-25.3]	8.975[0.699-30.1]	6.51[0.382-25.2]	11.2[0.311-39.2]	10.15[0.331-25.4]	0.969
PCT (ng/mL)	1.6[0.13-42.25]	0.75[0.07-100]	1.85[0.1-100]	0.58[0.14-100]	0.34[0.02-15.94]	0.72[0.11-20.37]	0.060
SAA (ng/mL)	45.52[0.38-77.03]	33.62[0.12-60.92]	39.79[2.54-61.21]	31.98[0.34-71.26]	18.52[0.29-58.63]	38.53[2.6-65.21]	0.315
**UA Nitrite**	1[1-2]	**1[c,d,f][1-2]**	1[1-1]	1[1-1]	1[1-2]	1[1-1]	**0,007**
**UA RBC**	**0[b] [0-3]**	5[0-728]	1[0-2]	3[0-36]	0[0-11]	0[0-87]	**0.010**
**UA WBC**	1[0-6]	**105[a,c,d,e,f] [4-562]**	1[0-5]	1[0-370]	1[0-84]	1[0-58]	**<0.001**
**Hospital stay (day)**	12[5-91]	11[4-51]	5[1-47]	12[1-56]	**6[a,b][1-20]**	5[2-17]	**0.005**

ALP: Alkaline Phosphatase; ALT: Alanine Aminotransferase; AST: Aspartate Aminotransferase; BUN: Blood Urea Nitrogen; CRP: C-Reactive Protein; DBP: Diastolic Blood Pressure; GGT: Gamma-Glutamyl Transferase; HB: Hemoglobin; HCT: Hematocrit; IL-6: Interleukin 6; LDH: Lactate Dehydrogenase; LY: Lymphocyte; MPV: Mean Platelet Volume; NE: Neutrophil; O_2: Oxygen Saturation; PCT: Procalcitonin; pH: Potential of Hydrogen; PLT: Platelet; SAA: Serum Amyloid A; SBP: Systolic Blood Pressure; UA Nitrite: Urinalysis Nitrite; UA RBC: Urinalysis Red Blood Cell; UA WBC: Urinalysis White Blood Cell; WBC: White Blood Cell.

a: There is a significant difference compared to cholangitis (p<0.05),

b: There is a significant difference compared to urinary tract infection (p<0.05),

c: There is a significant difference compared to sepsis ($p<0.05$),
d: There is a significant difference compared to other abdominal infections ($p<0.05$),
e: There is a significant difference compared to respiratory tract infection ($p<0.05$),
f: There is a significant difference compared to others ($p<0.05$).

Supplementary Table S5: Percentage* of final diagnoses within the main complaint in immunosuppressed patients presenting.

	Final Diagnosis					
Complaint at ED admission	Cholangitis	Urinary Tract Infection	Sepsis	Other Abdominal Infection	Respiratory System Infection	Other
Shortness of breath	%0	%0	%15	%5	%65	%15
Burning in urine, chills, shivering	%8.3	%66.7	%0	%25	%0	%0
General condition disorder, weakness	%0	%0	%50[c]	%6.3	%12.4	%31.3
Abdominal pain, fever	%50	%11.1	%0	%27.8	%0	%11.1
Nausea, vomiting, other	%28.6	%28.6	%7.1	%7.1	%14.3	%14.3

*: Percentage was calculated based on row totals.
